# Short-term responsiveness of DNA methylation–based aging biomarkers to a multimodal intervention comprising exercise and dietary guidance involving daily consumption of yogurt containing *Bifidobacterium longum* BB536: an exploratory randomized controlled trial

**DOI:** 10.18632/aging.206386

**Published:** 2026-05-29

**Authors:** Tatsuki Nishimura, Ayako Horigome, Miyuki Tanaka, Yukihiro Hishida, Toshitaka Odamaki

**Affiliations:** 1 Biotics Research Institute, R&D Division, Morinaga Milk Industry Co., Ltd., Zama, 252-8583, Japan; 2 Food Function Research Institute, R&D Division, Morinaga Milk Industry Co., Ltd., Zama, 252-8583, Japan

**Keywords:** DNA methylation clock, DunedinPACE, Multicomponent lifestyle intervention, *Bifidobacterium longum* BB536

## Abstract

Aging-related chronic diseases are driven by multiple mechanisms, motivating efforts to develop feasible interventions that can attenuate biological aging. DNA methylation-based epigenetic clocks, particularly measures of the pace of aging such as DunedinPACE, are sensitive to relatively short-term changes in aging processes. However, evidence from randomized controlled trials remains limited. We conducted a randomized controlled trial to test a 12-week multimodal lifestyle intervention comprising exercise and dietary guidance involving daily consumption of yogurt containing *Bifidobacterium longum* BB536 on DNA methylation-based aging measures in overweight men aged ≥50 years. The intervention group exhibited a significant deceleration in DunedinPACE, corresponding to an estimated 2.2% slower pace of aging, whereas no meaningful change was observed in the control group. Exploratory analyses further identified a significant reduction in DNAmCystatinC, a renal-related GrimAge surrogate marker, while no clock within the biological age remained significant after false discovery rate correction. These findings suggest that a feasible, multimodal lifestyle intervention—including exercise and dietary guidance with daily consumption of yogurt containing *Bifidobacterium longum* BB536—may be associated with short-term changes in selected DNA methylation-based aging measures. Larger and longer-term studies are warranted to confirm the durability and clinical relevance. This clinical trial was registered with the University Hospital Medical Information Network Clinical Trials Registry (UMIN000057293).

## INTRODUCTION

Aging is the predominant risk factor for chronic diseases such as cardiovascular disorders, diabetes, and neurodegeneration, positioning it as a primary target for preventive strategies [1]. The geroscience hypothesis suggests that interventions addressing fundamental aging mechanisms could simultaneously delay multiple age-related disorders and extend health span [2–4]. Reliable biomarkers are indispensable for assessing whether such interventions can modulate biological aging.

Epigenetic clocks have emerged as promising biomarkers of biological aging, capturing variation in morbidity, mortality, and functional decline that is not explained by chronological age alone [5, 6]. These algorithms are constructed by modeling DNA methylation patterns at specific CpG sites to generate composite indices that estimate biological age, or, in some cases, the pace of aging [6]. First-generation clocks, such as the Horvath and Hannum models, were trained to predict chronological age [7, 8], whereas second-generation clocks, including PhenoAge, GrimAge and GrimAge version 2 (GrimAge v2), were explicitly optimized against physiological biomarkers and mortality risk [9–11]. More recently, DunedinPACE was developed to quantify the pace of aging, and accumulating evidence suggests that it may be particularly sensitive to short-term biological responses to lifestyle modification compared with traditional age-prediction clocks [12–14].

Contemporary geroscience frameworks conceptualize aging as the cumulative consequence of multiple “hallmarks of aging”, encompassing chronic inflammation and deregulated nutrient-sensing, among others [3, 15]. These hallmarks progress in parallel and reinforce each other, making single-pathway interventions unlikely to produce substantial improvements. Indeed, obesity exemplifies a clinical state in which several hallmarks of aging—chronic low-grade inflammation and metabolic dysregulation—are concurrently perturbed, and higher BMI is consistently associated with accelerated epigenetic aging across several DNA methylation clocks [16–18]. In this context, lifestyle components have garnered attention because they have partially shared effects across diet and exercise. Dietary modifications primarily influence nutrient-sensing and mitochondrial integrity [19], whereas physical activity enhances mitochondrial efficiency while attenuating chronic inflammation [20]. Importantly, inflammation and nutrient-sensing can respond within weeks to coordinated changes in diet and physical activity. Accordingly, multicomponent lifestyle approaches, by engaging multiple hallmarks concurrently, are hypothesized to produce integrated biological effects detectable over relatively short timeframes, and to yield measurable deceleration in aging-related biomarkers compared with single-modality approaches. Using a biomarker that captures the current pace of aging, such as DunedinPACE, may increase the likelihood of detecting these early signals in the context of short-term lifestyle intervention.

Although interest in lifestyle interventions to promote healthy aging is increasing, randomized controlled trials and post hoc analyses evaluating their impact on pace of aging quantified by DunedinPACE remain limited [21]. In the CALERIE trial, a two-year caloric restriction intervention in non-obese healthy adults significantly slowed the pace of aging as measured by DunedinPACE, yet failed to alter biological age estimated by PhenoAge or GrimAge [13]. Conversely, in the DO-HEALTH trial of community-dwelling older Europeans receiving vitamin D, omega-3 fatty acids, and a home exercise program for three years, omega-3 supplementation alone induced small but statistically significant reductions in PhenoAge, GrimAge v2, and DunedinPACE, whereas vitamin D or exercise alone showed no clear effects on any clock, although an additive benefit was observed only for PhenoAge when all three interventions were combined [22]. On the other hand, comparatively short- or mid-term trials were limited. Small pilot studies with multiple lifestyle programs, including diet, exercise, stress management, and supplementation with *Lactobacillus plantarum* 299v, were associated with a short-term reduction in salivary Horvath clock, although DunedinPACE was not measured [16, 17]. Conceptually, probiotics are often discussed as multi-component interventions because they comprise diverse microbial-derived factors, such as cell wall components and metabolites, which may collectively interact with host pathways related to multiple hallmarks of aging [23]. From a theoretical perspective, such characteristics could be hypothesized to support the detection of integrated biological responses in short-term intervention studies, although this remains to be empirically validated. This clock- and intervention-dependent variability underscores the complexity of aging processes and highlights the necessity of integrating multiple epigenetic biomarkers and studies to capture robust effects. Nevertheless, short- to mid-term randomized evidence on responsiveness of epigenetic aging measures remains limited [18].

Indeed, a recent cross-sectional analysis reported that adherence to a healthy dietary pattern was associated with slower biological aging, as estimated by multiple DNA methylation clocks, in older adults, although the evidence remains observational [24]. Therefore, we conducted an exploratory randomized controlled trial in overweight men to examine whether a pragmatic multicomponent lifestyle intervention could induce detectable short-term changes in DNA methylation–based measures of biological aging. The intervention combined individualized physical activity and dietary counseling with daily intake of yogurt containing *Bifidobacterium longum* BB536 (BB536). Based on prior reports suggesting that BB536 has multiple components relevant to aging-related pathways (e.g., inflammatory and oxidative stress signaling) [25, 26], it was included as one element from food components of a multicomponent lifestyle intervention intended to target multiple aging-related pathways concurrently.

## RESULTS

Male participants aged 50-74 years were recruited and randomized to the intervention group (INT, *n* = 24) or control group (CON, *n* = 24) using predefined allocation factors (chronological age and BMI) as shown in Figure 1. Per-protocol analyses were conducted in INT (*n* = 21) and CON (*n* = 24). Baseline characteristics of each group are summarized in Table 1, and participant-level baseline values are provided in Supplementary Table 4. No significant differences were observed between the two groups in chronological age, body weight, BMI, or smoking habits. During intervention, all participants in the INT group complied with exercise guidance (Supplementary Table 1). As the intervention incorporated diet and exercise counseling, an effect on body weight was anticipated. Indeed, following the intervention, body weight and BMI were significantly reduced in the INT group (Table 2).

**Figure 1:**
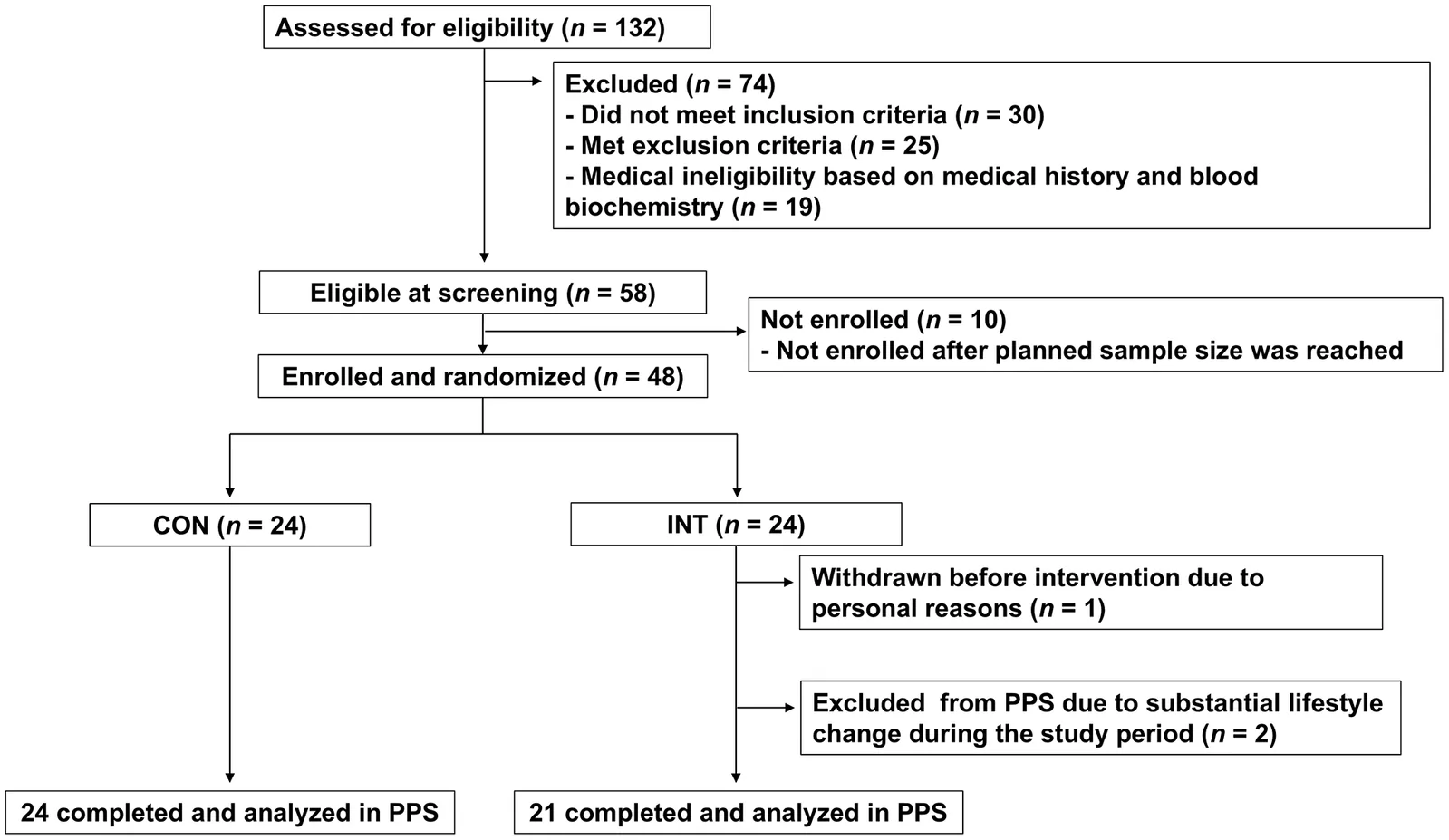
**CONSORT flow diagram of the study participants.** The flow diagram illustrates the progress of participants through the phases of the trial. Forty-eight participants were randomized (INT, *n* = 24; CON, *n* = 24); in the INT group, one participant withdrew for personal reasons and two were excluded because unintended lifestyle changes introduced after randomization, resulting in per-protocol samples (PPS) of 21 in the INT group and 24 in the CON group, respectively.

**Table 1:** Baseline characteristics of subjects.

	**INT (*n* = 21)**	**CON (*n* = 24)**	***p*-value**
Chronological age (years), mean ± SD	57.38 ± 4.36	57.96 ± 5.27	0.690^a^
Body weight (kg), mean ± SD	83.42 ± 7.83	81.11 ± 8.34	0.345^a^
BMI (kg/m^2^), mean ± SD	27.90 ± 2.78	27.66 ± 2.50	0.761^a^
Current smoker, n (%)	4 (19.0)	8 (33.3)	0.329^b^
Sleep duration (h), mean ± SD	6.47 ±1.02	6.46 ±1.03	0.954^a^

^a^*p*-values determined by a two-sample t test, ^b^*p*-values determined by Fisher’s exact test.

**Table 2:** Body weight, BMI and POMS 2 TMD score (mean ± SD.) at before and after intervention.

	**Group**	**Week 0**	**Week 12**	***p*-value (vs. Week 0) ^b^**
Body weight (kg)	INT (*n* = 21)	83.42 ± 7.83	80.50 ± 7.44	<0.001
	CON (*n* = 24)	81.11 ± 8.34	80.74 ± 8.24	0.384
	*p*-value^a^	0.345	0.922	
BMI (kg/m^2^)	INT (*n* = 21)	27.90 ± 2.78	26.91 ± 2.51	<0.001
	CON (*n* = 24)	27.66 ± 2.50	27.53 ± 2.44	0.394
	*p*-value^a^	0.761	0.406	
POMS 2 TMD score	INT (*n* = 21)	44.14 ± 5.42	44.14± 6.90	1.000
	CON (*n* = 24)	45.13± 8.39	46.33 ± 7.79	0.217
	*p*-value^a^	0.649	0.327	

^a^*p*-values determined by a two-sample t test, ^b^*p*-values determined by paired t-test.

To explore how the intervention might influence DNA methylation–based aging biomarkers across multiple dimensions, we examined changes in the pace of aging using DunedinPACE, alongside changes in current estimation of biological age (PC-HannumClock, PC-HorvathClock, PC-PhenoAge, GrimAge, and GrimAge v2) and GrimAge-derived surrogate markers. For DunedinPACE the adjusted mean change was −0.022 (95%CI −0.039 to −0.004) in the INT group and +0.001 (95%CI −0.015 to +0.017) in the CON group, resulting in a modest but statistically significant between-group difference of −0.023 (95%CI −0.045 to −0.001; *p* = 0.045; Figure 2A). Given that DunedinPACE is a pace of aging measure with 1.0 indicating approximately one biological year per chronological year, this corresponds to an approximately 2.2% slowing in the pace of aging within the INT group and a 2.3% slower pace of aging relative to controls over 12 weeks. Within the biological age family, no clock remained statistically significant after false discovery rate (FDR) correction. Detailed results for all biological age and raw values are shown in Supplementary Tables 1, 3, respectively. Prior to FDR adjustment, GrimAge showed a nominal between-group difference (*p* = 0.049; Figure 2B), but this did not withstand correction for multiple testing. No significant associations were observed between changes in DNA methylation–based aging measures and body weight reduction (Figure 3).

**Figure 2:**
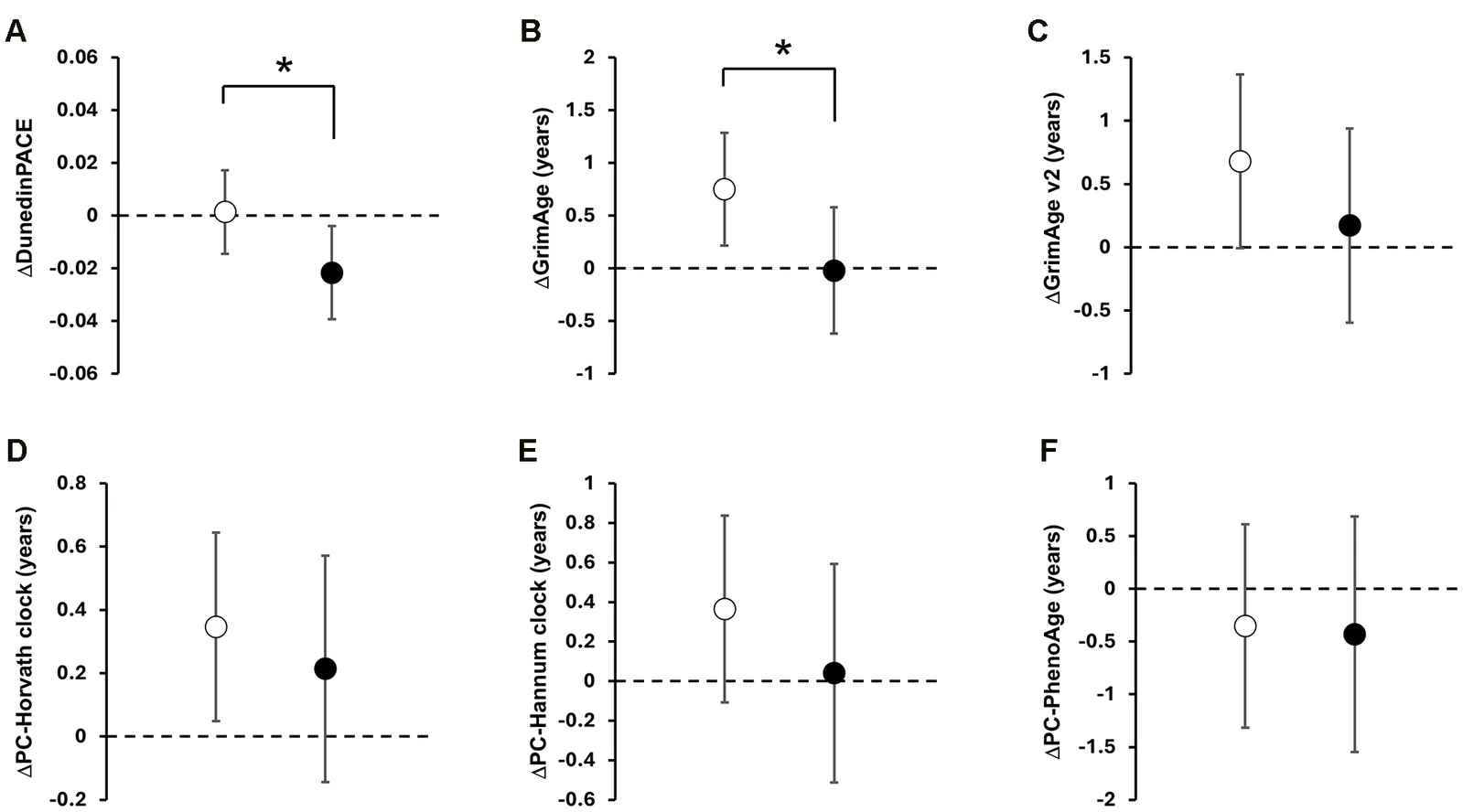
**Effect of the multimodal lifestyle intervention on DNA methylation-based clocks.** Data are presented as adjusted mean changes from baseline with 95% confidence intervals (CI). Open and black circles represent an adjusted mean change in the CON and INT group, respectively. Scale bars show 95% CI. The dotted line indicates zero. Asterisks (*) indicate significant between-group differences by ANCOVA (*p* < 0.05). Panels show; (**A**) DunedinPACE; (**B**) GrimAge; (**C**) GrimAge v2; (**D**) PC-Horvath clock; (**E**) PC-Hannum clock; (**F**) PC-PhenoAge.

**Figure 3:**
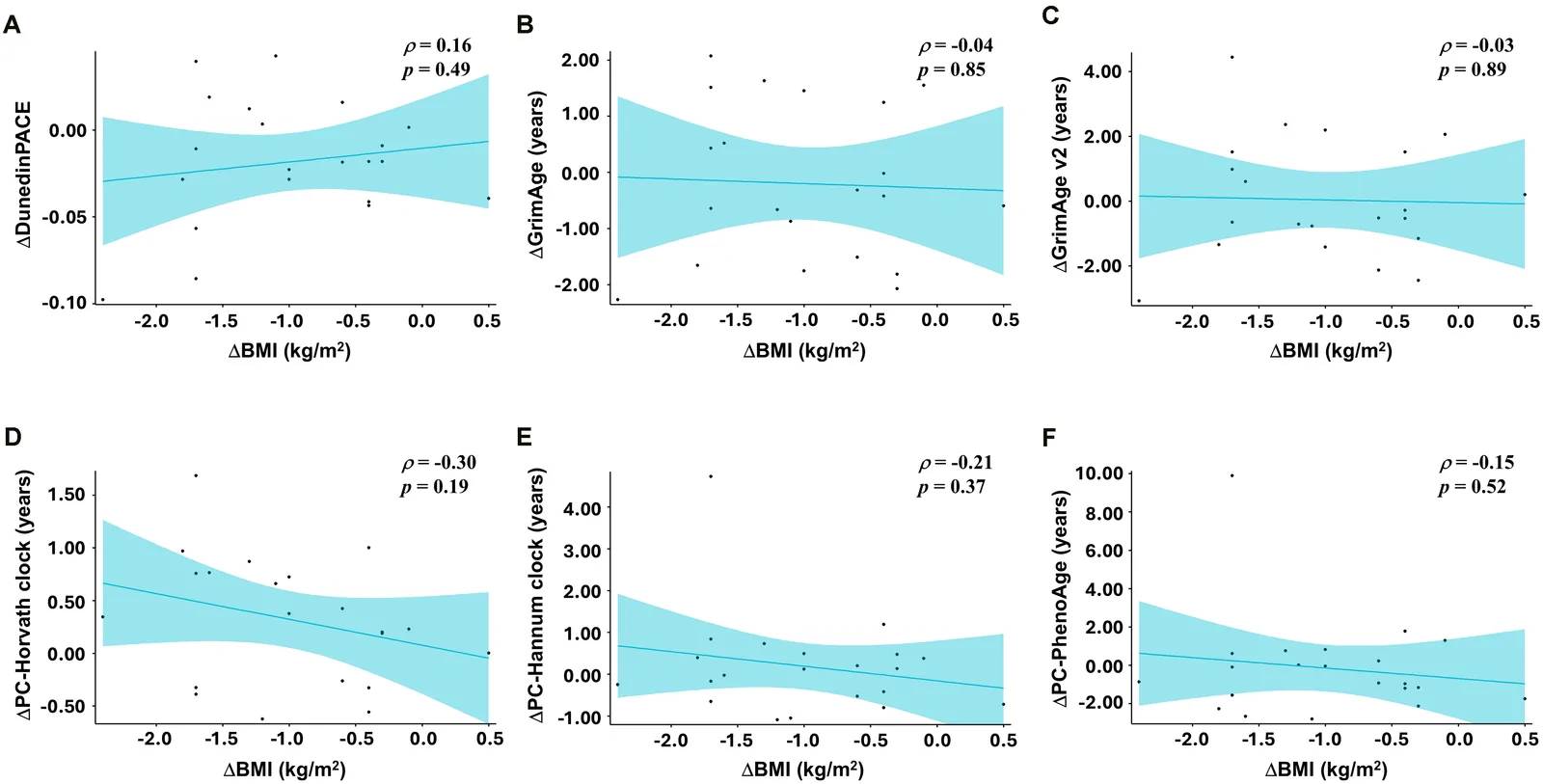
**Correlation between changes in body weight and DNAm-based aging measures.** Scatter plots illustrating associations between changes in body mass index (ΔBMI, kg/m^2^) and changes in DNA methylation-derived age acceleration (week 12 minus baseline) in the INT group (*n* = 21). Each point represents an individual participant; solid lines depict simple linear regression fits, shown for visualization only. Pearson correlation coefficients (*p*) and corresponding p-values are shown in each panel. Panels show; (**A**) ΔDunedinPACE vs. ΔBMI; (**B**) ΔGrimAge vs. ΔBMI; (**C**) ΔGrimAge v2 vs. ΔBMI; (**D**) ΔPC-Horvath clock vs.ΔBMI; (**E**) ΔPC-Hannum clock vs. ΔBMI; (**F**) ΔPC-PhenoAge vs. ΔBMI.

In exploratory analyses of GrimAge-derived surrogate markers, including DNAm-based estimators of plasma proteins (e.g., DNAmCystatinC, DNAmADM, DNAmPAI-1, DNAmGDF15), DNAmCystatinC showed a significant between-group difference and remained significant after FDR correction (*p* = 0.001; *q* < 0.01; Figure 4A). Detailed results for all GrimAge-derived surrogate markers and raw values are provided in Supplementary Tables 2, 3, respectively.

**Figure 4:**
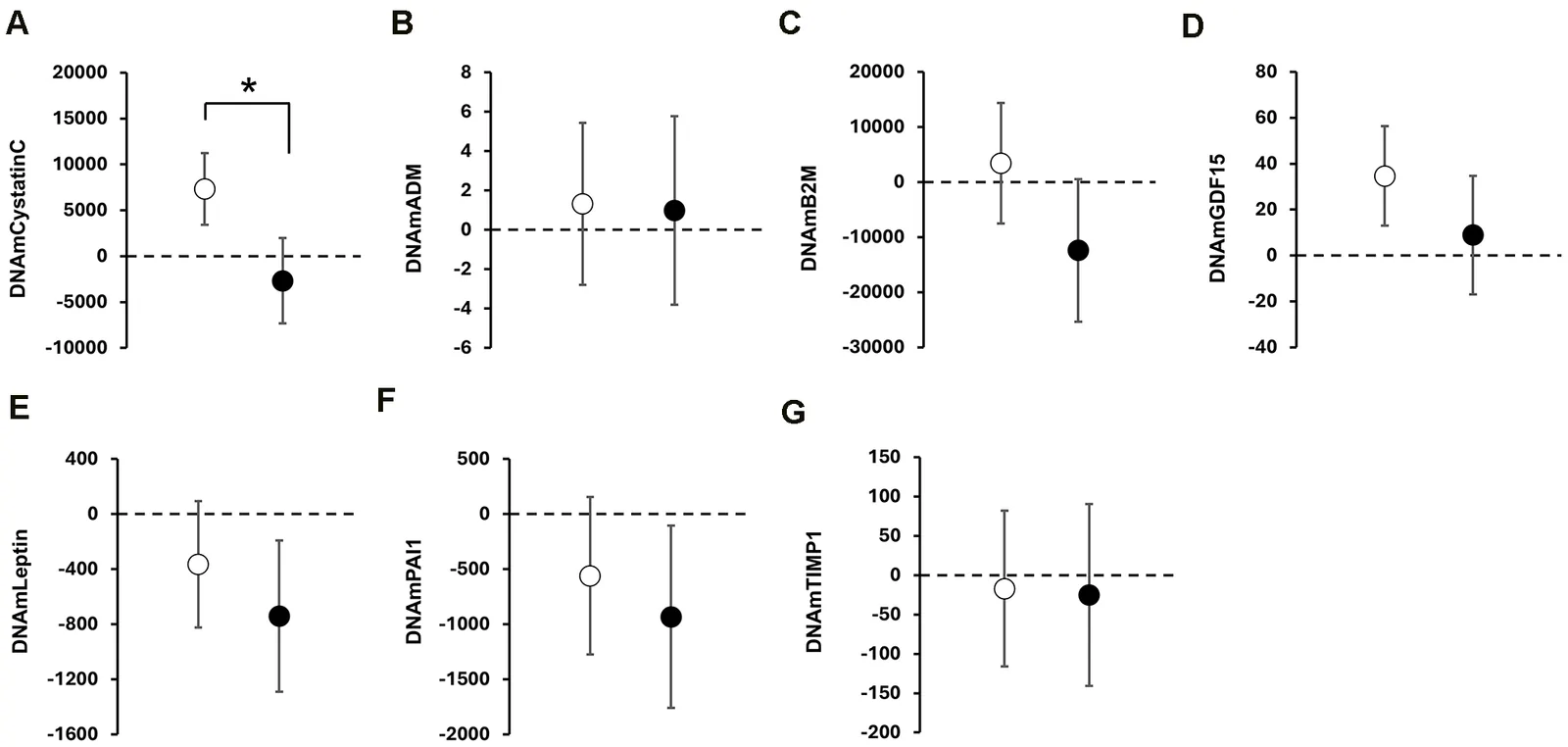
**Exploratory analyses of DNAm-based surrogate biomarkers of GrimAge.** Data are presented as adjusted mean value in DNAm-based surrogate biomarkers of GrimAge with 95% confidence intervals (CI) at post-intervention. Open and black circles represent an adjusted mean value in the CON and INT group, respectively. Scale bars show 95% CI. The dotted line indicates zero. Asterisks (*) indicate significant between-group differences by ANCOVA. Panels show; (**A**) DNAmCystatinC; (**B**) DNAmADM; (**C**) DNAmB2M; (**D**) DNAmGDF15; (**E**) DNAmLeptin; (**F**) DNAmPAI1; (**G**) DNAmTIMP1.

## DISCUSSION

This exploratory randomized controlled trial showed that a multicomponent lifestyle intervention combining dietary guidance, physical activity counseling, and daily intake of yogurt containing BB536 was associated with short-term change in DNA methylation-based aging biomarkers in overweight, middle-aged to older men. Specifically, DunedinPACE, a measure of the current pace of aging that has been prospectively associated with risk of mortality and cardiovascular disease, showed a modest but nominally statistically significant between-group difference over 12 weeks. In contrast, no clock within the biological-age family remained significant after FDR correction. Exploratory analyses of GrimAge-derived surrogate markers identified reduction in DNAmCystatinC as a supportive finding.

In the control group, DunedinPACE changed minimally (+0.001 units, ≈+0.1%), consistent with expected natural drift and the high test–retest reliability of this measure [12]. By contrast, the ≈2.2% slowing of DunedinPACE in the INT group over 12 weeks is modest but within the same order of magnitude as the ≈2–3% slowing reported over 2 years of 25% caloric restriction in the CALERIE trial and the ≈3 months of protection observed over 3 years of omega-3 supplementation in DO-HEALTH across multiple clocks, including DunedinPACE [13, 22]. These comparisons suggest that, if sustained, the magnitude of short-term changes observed here is within the range previously associated with longer-term lifestyle interventions. However, their clinical relevance requires confirmation in adequately powered, long-term trials.

Our findings align with prior evidence linking adverse cardiometabolic profiles to accelerated epigenetic aging, particularly as captured by DunedinPACE. Higher adiposity, metabolic syndrome, and pro-inflammatory dietary patterns have been associated with DunedinPACE acceleration, often exceeding associations observed for other DNA methylation clocks [23], whereas adherence to anti-inflammatory dietary patterns and favorable cardiovascular risk profiles has been associated with slower DunedinPACE [27, 28]. Within this context, the DunedinPACE deceleration observed here may, at least in part, reflect the combined effects of (i) optimized metabolic regulation and reduced lipotoxicity, (ii) enhanced insulin sensitivity, vascular function, and muscle maintenance via regular exercise, and (iii) improved gut homeostasis and lower systemic inflammation. Importantly, DunedinPACE deceleration in the INT group was not statistically correlated with the BMI reduction (Figure 3). In exploratory analysis, the weekly frequency of exercise sessions was not associated with DunedinPACE (Supplementary Figure 1). However, because energy intake, diet quality, or expenditure were not quantified using validated instruments, and exercise intensity/volume (e.g., METs-minutes) was not measured, mediation analyses and interpretation of diet- or activity-linked effects are limited. Taken together, these observations suggest that neither weight loss alone nor exercise habits alone fully account for the observed slowing of biological aging. We hypothesize that additive or integrated modulation of systemic inflammation through microbiota-derived anti-inflammatory metabolites such as tryptophan-derived metabolites [29] and SCFAs [30], alongside diet [31] and exercise [32], may contribute, even if individual main effects are not detectable.

Among the DNAm-based surrogate protein components constituting GrimAge, the renal function-related marker DNAmCystatinC showed a significant between-group difference, with a clear decrease in the intervention group relative to controls, and remained significant after FDR correction within the surrogate-marker family (*p* = 0.001; *q* < 0.01, Figure 4A and Supplementary Table 3). Directionally similar but nonsignificant change was also observed in DNAmB2M (Supplementary Table 3), which is incorporated into the GrimAge algorithm as a surrogate for glomerular filtration [10]. GrimAge showed only a nominal between-group difference, but this did not remain significant after FDR correction. These findings may suggest that the observed signals were more localized to renal-related components within the GrimAge framework than broadly reflected across composite epigenetic age measures. Accordingly, the decrease in DNAmCystatinC, together with the directionally similar change in DNAmB2M, may indicate reduced physiological renal stress.

Taken together, the observed decrease in DNAmCystatinC may be interpreted as a supportive signal within a renal- and metabolic-related physiological domain [33, 34]. One biological hypothesis for future investigation is that microbiota-related alterations in indole metabolism may contribute to this domain, as suggested by the potential roles of indoxyl sulfate and indole-3-lactic acid (ILA). Indole, produced by gut microbiota from tryptophan, is converted in the liver to indoxyl sulfate—a compound that promotes chronic kidney disease through oxidative stress, inflammation, and fibrosis—whereas Bifidobacterium species, including BB536, can convert indole into ILA, a beneficial metabolite with anti-inflammatory and potentially renoprotective properties [26, 35–38]. As this study assessed only DNA methylation-based surrogate markers, the underlying biological mechanisms remain unresolved. Future investigations employing transcriptomic and microbiota analyses will be critical to clarify these mechanisms.

From a translational standpoint, it is noteworthy that the dietary component of our intervention emphasized moderating overeating based on daily food records rather than imposing stringent caloric restriction. Adherence to the prescribed exercise program was excellent, with participants completing an average 5.2 sessions per week and thereby achieving approximately 156 min per week, which meets the lower threshold of WHO 2020 recommendation for adults to maintain their health [39] (Supplementary Table 5). Further, psychological stress appears to be linked to epigenetic age acceleration in prior studies [16, 17, 40], raising concern that intensive lifestyle programs could paradoxically attenuate benefits via stress-related pathways. In our trial, however, Profile of Mood States 2 (POMS 2) total mood disturbance scores did not change within or between groups (Table 2), suggesting minimal psychological burden. Notably, although participants did not habitually consume yogurt at breakfast before the trial, adherence to daily BB536-containing yogurt intake was high (≥99%), indicating that this single dietary change was acceptable and sustainable over the intervention period. Taken together, these observations suggest that modest adjustments in diet and physical activity, combined with a realistic and easily adoptable habit of daily BB536-containing yogurt consumption, may constitute a feasible lifestyle strategy for inducing short-term changes in biomarkers related to the pace of biological aging, a hypothesis that warrants confirmation in larger, long-term trials.

Several limitations should be acknowledged. First, because the intervention combined dietary counseling, exercise, and BB536-containing yogurt, the present study cannot attribute the observed epigenetic changes to any single component or their interaction. Accordingly, the findings should be interpreted at the level of the intervention package. Second, this trial was exploratory in nature and was not powered or pre-registered to test a single primary epigenetic endpoint. Accordingly, observed patterns across multiple clocks and DNAm surrogates should be regarded as hypothesis-generating rather than definitive. Third, the relatively small sample size (*n* = 45) and short intervention duration (12 weeks) limit statistical power and preclude conclusions regarding long-term sustainability of epigenetic changes or clinical outcomes. Although baseline differences in DNA methylation-based measures were adjusted for in the ANCOVA analyses, the small sample size means that some residual imbalance may persist despite randomization. Furthermore, the durability of the observed short-term changes awaits confirmation in future studies, which will be important for clarifying the long-term impact of such interventions. Fourth, the study population consisted exclusively of overweight Japanese men aged 50–74 years, restricting generalizability to women, younger individuals, or other ethnic groups. Previous research in a Western population has suggested that similar multimodal lifestyle interventions could reduce epigenetic aging in both men and women [16, 17], although these trials used different epigenetic clocks and study designs. Future studies should therefore explicitly include female participants to confirm broad applicability. Fifth, given the open-label design, blinding was not feasible, and participants’ awareness of group assignment may have led to additional unmeasured health behavior changes in the INT group. Finally, although the data suggest several hypotheses, mechanistic pathways—such as the role of *Bifidobacterium*-derived metabolites in modulating systemic inflammation and epigenetic aging—remain speculative and cannot be evaluated within the present design, as noted above. Future research may also benefit from complementing DNA methylation-based measures with RNA-based transcriptomic aging measures, which may be more responsive to rapid biological changes in short-term trials. Large-scale, long-duration randomized trials will then be required to assess the durability of epigenetic changes, active components, and their clinical relevance.

## MATERIALS AND METHODS

### Study design and adherence

This exploratory, randomized, open-label, parallel-controlled trial evaluated the short-term responsiveness of multiple DNA methylation–based aging biomarkers to a multimodal lifestyle intervention. The study did not include a single prespecified primary endpoint. The trial was conducted at the Nihonbashi Cardiology Clinic in Tokyo, Japan, from June to August 2025. Male adults aged 50-74 years who met the eligibility criteria were enrolled. As shown in Figure 1, a total of 132 individuals were screened for eligibility. Of these, 30 did not meet the inclusion criteria, 25 met one or more exclusion criteria, and 19 were judged medically ineligible based on physician assessment of medical history and blood biochemistry results. Consequently, 58 individuals were deemed eligible. Because the planned enrollment target for the trial was 48 participants, eligible candidates were approached sequentially to confirm their willingness to participate, and enrollment continued until the target sample size was reached. Randomization was performed by an independent investigator using stratified block randomization based on age and body mass index (BMI), assigning participants to either the intervention group (INT), which received a structured exercise and dietary counseling program along with the test food, or the control group (CON), which was instructed to maintain their habitual lifestyle throughout the study. Given the exploratory nature of the trial, sample size was determined by the maximum feasible enrollment at the study site within operational constraints rather than formal power calculation. The study comprised a 1-week pre-observation period followed by a 12-week intervention period, during which adherence was monitored by recording daily steps with a pedometer and tracking body weight and dietary intake, as detailed in the respective measurement sections. Participants also recorded daily intake of the test food, changes in physical condition or lifestyle, use of medications or quasi-drugs, engagement in walking or stepping exercises from baseline (week 0) through week 12.

### Test food

Participants in the INT group consumed one serving (100 g) of yogurt (Bifidus plain yogurt; Morinaga Milk Industry Co., Ltd., Tokyo, Japan) containing *Bifidobacterium longum* BB536 (2 × 10^9^ CFU per 100 g) once daily after breakfast. Participants in the CON group did not receive any test food.

### Dietary counseling

Individualized dietary counseling for the INT group was based on simplified daily dietary records maintained throughout the intervention. Counseling was provided at clinic visits (weeks 0, 2, 4, and 8) and by telephone (weeks 1, 3, 5, 6, 9 and 10). Sessions focused on gradual weight reduction through moderation of overall energy intake and limiting snacks and sugar-sweetened beverages, rather than imposing strict caloric quotas. Participants in the CON group were instructed to maintain their habitual dietary patterns via the study period.

### Exercise counseling

Participants in the INT group were advised to engage in walking or stepping exercises (moderate-to-vigorous physical activity) using a stepper device (Mini Stepper BDA001BK; WEIWEI Holdings Co., Ltd., Fukuoka, Japan) for a total of 30 minutes per day on at least 3 days per week. Exercise counseling was provided at the same time points as dietary counseling and was based on participants’ self-recorded exercise logs. Participants in the CON group were instructed to maintain their habitual exercise patterns during the study.

### Participants

Forty-eight eligible overweight male participants met the inclusion criteria and were enrolled. One participant withdrew during the pre-observation period. Eligibility was determined according to the inclusion and exclusion criteria. The inclusion criteria were as follows: (1) male, aged ≥50 years and <75 years at consent; (2) BMI ≥25 kg/m^2^; (3) no habitual intake of fermented milk products (≤1 time/week); (4) medically cleared for regular exercise; and (5) ability to measure daily step count. The study was limited to men to avoid potential confounding from variation in female sex hormone status across a similar age range. The exclusion criteria were as follows: (1) current or past serious diseases (e.g., malignant tumors, respiratory disorders, or significant hepatic, renal, cardiac, pulmonary, gastrointestinal, or metabolic illnesses); (2) current or past intestinal diseases; (3) severe drug or food allergies; (4) lactose intolerance; (5) regular use of medications affecting bowel movements (e.g., laxatives, intestinal regulators, or purgatives); (6) antibiotic use >1 week within the past 3 months; (7) inability to discontinue the consumption of specific health foods, functional dietary products, nutritional supplements, or foods and beverages containing bifidobacteria, lactic acid bacteria, oligosaccharides, or lactoferrin during the study period; (8) active dietary restriction or weight-loss programs including participation; (9) habitual meal skipping; (10) current exercise, dietary, or health guidance under medical supervision; (11) moderate or more vigorous exercise ≥30 min, ≥3 times/week (excluding commuting); (12) heavy smoking (≥21 cigarettes/day); (13) alcohol intake >60 g/day; (14) blood donation >200 mL within 1 month or >400 mL within 3 months prior to screening; (15) irregular lifestyle (e.g., night shifts, frequent irregular work shifts, or physically demanding labor); (16) anticipated lifestyle changes during the study; (17) participation in another clinical trial within the past month, or intent to participate; and (18) any other condition deemed inappropriate by the principal investigator.

### Sample collection

Whole blood samples were collected after an overnight fast at baseline (week 0) and at the end of the 12-week intervention (week 12) using PAXgene DNA tubes (QIAGEN N.V., Hilden, Germany) and stored at 4° C until DNA methylation analysis.

### Body weight and body fat percentage measurement

Participants recorded their body weight at home using a personal scale. These daily values, together with dietary records, were used to inform counseling. Body weight and body fat percentage were formally assessed at each clinic visit (screening, baseline [week 0], weeks 2, 4, 8, and 12) after an overnight fast using a multifrequency bioelectrical impedance analyzer (InBody 470; InBody Japan, Inc., Tokyo, Japan). BMI was calculated from height measured at week 0.

### Dietary assessment

To support and monitor the dietary intervention, participants in the INT group completed a simplified daily dietary record from the 1-week pre-observation period through the intervention. They documented all food and beverage consumed, including approximate quantities, for breakfast, lunch, dinner, as well as snacks, and alcohol drinks. These self-reported records were used to assess adherence to the prescribed dietary guidance and to refine subsequent individualized counseling.

### POMS 2 assessment

Mood states were assessed using the Profile of Mood States Second Edition – Short Form (POMS 2-SF) (Kaneko Shobo Co., Ltd., Tokyo, Japan) at week 0 and post-intervention (week 12), as previously described [41]. The questionnaire was self-administered by participants at home. This instrument evaluates six mood domains and a Total Mood Disturbance (TMD) score. Participants rated each item on a 5-point Likert scale (0 = “Not at all” to 4 = “Extremely”), based on their feelings during the past week, including the day of assessment. Scoring followed the POMS 2 manual, with raw scores converted to T-scores using age- and gender-adjusted norms.

### DNA methylation analyses

Genomic DNA from peripheral blood circulating leukocyte samples was purified using the Maxwell^®^ RSC Blood DNA Kit (Promega Corporation, Madison, WI, USA) according to the manufacturer’s instructions. The concentration of genomic DNA was quantified using the QuantiFluor^®^ ONE dsDNA System (Promega Corporation, Madison, WI, USA). A total of 250 ng of DNA was bisulfite-converted using the EZ-96 DNA Methylation Kit (Zymo Research, Irvine, CA, USA), and the converted DNA was used as input for the Infinium^®^ MethylationEPIC v2.0 BeadChip array (Illumina Inc., San Diego, CA, USA).

The EPIC arrays were processed according to the manufacturer’s protocol and scanned on an iScan System (Illumina Inc., San Diego, CA, USA). Beta values for each probe were extracted from IDAT files using the SeSAMe R package (https://bioconductor.org/packages/release/bioc/html/sesame.html, version 1.24.0), followed by normalization and removal of unsuitable probes.

### Measurement of epigenetic clocks and the eight DNAm-based protein surrogates constituting the GrimAge

Preprocessed DNA methylation profiles were provided by Rhelixa Inc. (Tokyo, Japan) and were used to assess epigenetic clocks and to estimate the seven DNAm-based protein surrogate markers for GrimAge. All epigenetic clocks, except DunedinPACE, were expressed as age acceleration (AgeAccel), calculated as the residual from a linear regression of DNAm-estimated age on chronological age. All DNAm-based protein surrogate markers were expressed as residual with age-adjusted levels. Principal component–based versions of the Horvath, Hannum, and PhenoAge clocks were computed using R scripts available on GitHub (https://github.com/MorganLevineLab/PC-Clocks/). GrimAge and its DNAm-based protein surrogates were calculated using coefficient files provided in the GrimAgeV1 dataset (https://github.com/bio-learn/biolearn/blob/master/biolearn/data/GrimAgeV1.csv). GrimAge v2 and its corresponding DNAm-based protein surrogates were computed using the GrimAgeV2 dataset (https://github.com/bio-learn/biolearn/blob/master/biolearn/data/GrimAgeV2.csv). DunedinPACE values were derived using the coefficient file from the DunedinPACE dataset (https://github.com/bio-learn/biolearn/blob/master/biolearn/data/DunedinPACE.csv).

### Statistical analysis

Given the exploratory design of the study and the absence of a prespecified primary endpoint, the primary efficacy population was the per-protocol set (PPS). Baseline characteristics were compared between groups using Fisher’s exact test for categorical variables and the two-sample t-test for continuous variables. For continuous variables, between-group differences were assessed using two-sample t-tests, and within-group changes from baseline to follow-up were evaluated using paired t-tests performed using IBM SPSS Statistics (version 29.0.2.0; IBM Corp., Armonk, NY, USA). PERMANOVA and Pearson correlation analyses were performed using R software (version 4.5.2). Pearson correlation coefficients were calculated using change-from-baseline values for body weight and the weekly frequency of moderate-to-vigorous physical activity, including sessions undertaken as part of the exercise counseling in this study. Intergroup comparisons of multiple DNA methylation–based aging clocks and GrimAge-derived DNA methylation surrogates were performed using analysis of covariance (ANCOVA) in IBM SPSS Statistics (version 29.0.2.0; IBM Corp., Armonk, NY, USA), with baseline values of each clock, chronological age, smoking habit, and sleep duration included as covariates. In an additional exploratory analysis, given the conceptual distinction between DunedinPACE and the DNAm age-acceleration clocks, multiplicity was handled using two endpoint families; pace-of-aging (DunedinPACE) and current biological age (PC-HorvathClock, PC-HannumClock, PC-PhenoAge, GrimAge, and GrimAge v2). False discovery rate (FDR) was controlled within the biological-age endpoint family using the Benjamini-Hochberg (BH) procedure. GrimAge surrogate markers were analyzed as a separate exploratory family, and FDR was likewise controlled using the BH procedure.

### Data availability

The datasets generated and/or analyzed during the current study, including processed DNA methylation data and derived epigenetic clock measures, are available from the corresponding author upon reasonable request, subject to ethical approval and applicable privacy regulations.

## Supplementary Material

Supplementary Figure 1

Supplementary Tables 1-3

Supplementary Table 4

Supplementary Table 5
