# Supplementary material for: Short-term responsiveness of DNA methylation–based aging biomarkers to a multimodal intervention comprising exercise and dietary guidance involving daily consumption of yogurt containing Bifidobacterium longum BB536: an exploratory randomized controlled trial
Source: Aging (Albany NY). 2026 May 29;18(1):639–55. doi: 10.18632/aging.206386 (PMC13632858; doi:10.18632/aging.206386)
Supplement: Supplementary file 1 [file aging-18-1-206386-s001.pdf]

SUPPLEMENTARY FIGURE

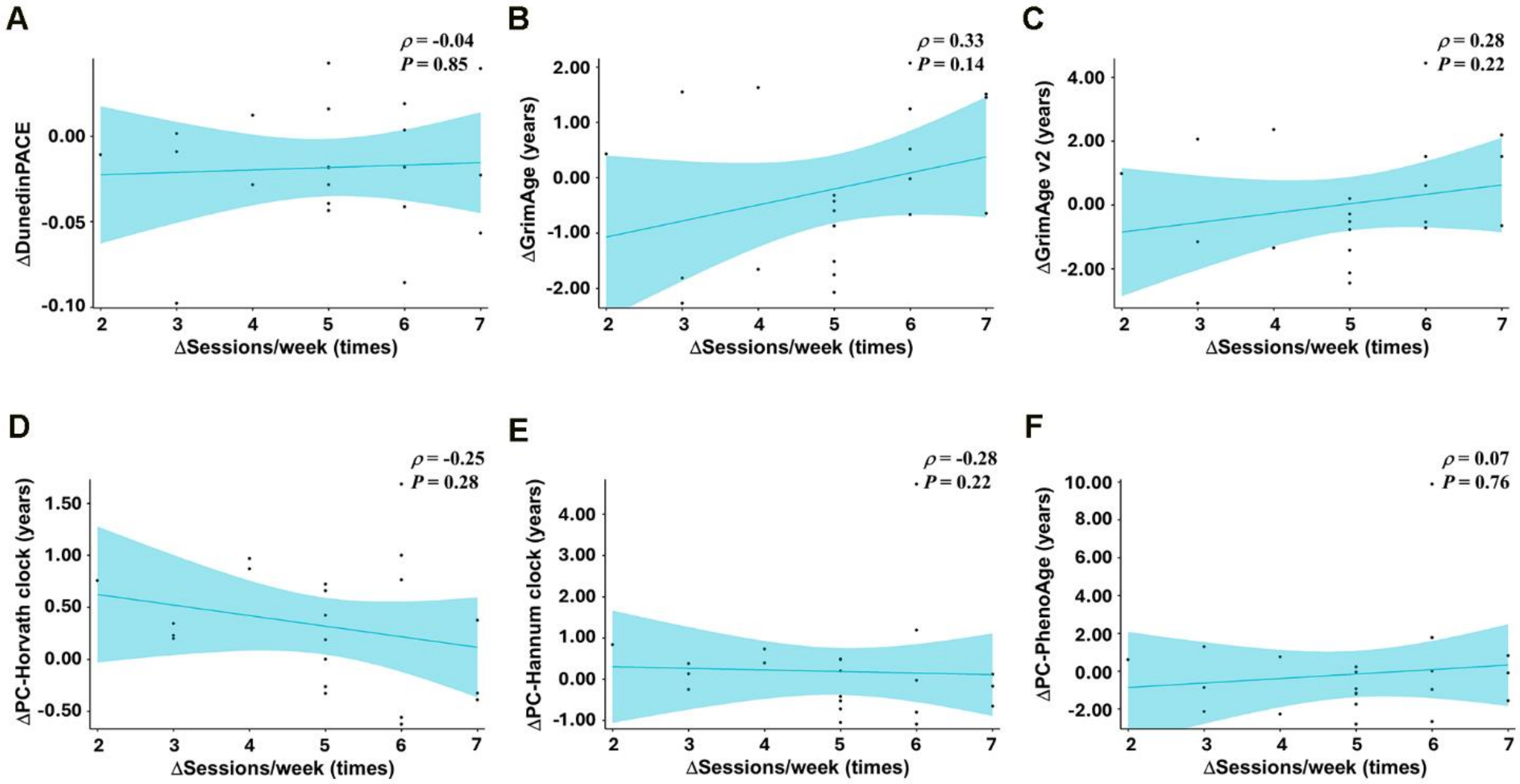

Supplementary Figure 1. Correlation between changes in frequency of exercise sessions and DNAm-based aging measures. Scatter plots illustrating associations between changes in exercise frequency (Δsessions/week; week 12 minus baseline) and changes in DNA methylation–derived age acceleration (week 12 minus baseline) in the INT group (n = 21). Each point represents an individual participant; solid lines depict simple linear regression fits, shown for visualization only. Pearson correlation coefficients (p) and corresponding p-values are shown in each panel. Panels show; (**A**) ΔDunedinPACE vs. Δsessions/week; (**B**) ΔGrimAge vs. Δsessions/week; (**C**) ΔGrimAge v2 vs. Δsessions/week; (**D**) ΔPC-Horvath clock vs. Δsessions/week; (**E**) ΔPC-Hannum clock vs. Δsessions/week; (**F**) ΔPC-PhenoAge vs. Δsessions/week.
