# Supplementary material for: Short-term responsiveness of DNA methylation–based aging biomarkers to a multimodal intervention comprising exercise and dietary guidance involving daily consumption of yogurt containing Bifidobacterium longum BB536: an exploratory randomized controlled trial
Source: Aging (Albany NY). 2026 May 29;18(1):639–55. doi: 10.18632/aging.206386 (PMC13632858; doi:10.18632/aging.206386)
Supplement: Supplementary file 2 [file aging-18-1-206386-s002.pdf]

SUPPLEMENTARY TABLES

Supplementary Table 1. Effect of the multimodal lifestyle intervention on DNA methylation-based aging markers.

Aging clock	INT			CON			p-value	q-value
	Estimated marginal mean	Lower confidence limit	Upper confidence limit	Estimated marginal mean	Lower confidence limit	Upper confidence limit		
GrimAge	-0.020	-0.618	0.578	0.750	0.214	1.286	0.049	0.245
GrimAge v2	0.173	-0.594	0.940	0.680	-0.006	1.367	0.304	0.590
PC-PhenoAge	-0.432	-1.549	0.685	-0.352	-1.318	0.614	0.910	0.910
PC-Horvath clock	0.214	-0.144	0.572	0.347	0.049	0.645	0.556	0.695
PC-Hannam clock	0.041	-0.512	0.593	0.365	-0.108	0.838	0.354	0.590

Supplementary Table 2. Effect of the multimodal lifestyle intervention on surrogate markers of GrimAge.

Surrogate marker	INT			CON			p-value	q-value
	Estimated marginal mean	Lower confidence limit	Upper confidence limit	Estimated marginal mean	Lower confidence limit	Upper confidence limit		
rawADM	0.984	-3.799	5.768	1.315	-2.804	5.434	0.911	0.911
rawB2M	-12414.536	-25329.874	500.803	3418.510	-7498.419	14335.440	0.052	0.182
rawCystatinC	-2683.015	-7329.264	1963.233	7316.781	3403.346	11230.216	0.001	0.007
rawGDF15	8.900	-16.951	34.751	34.670	12.985	56.355	0.121	0.282
rawLeptin	-740.730	-1290.489	-190.971	-364.892	-823.197	93.412	0.268	0.469
rawPAI1	-933.099	-1760.019	-106.179	-559.878	-1273.115	153.359	0.466	0.652
rawTIMP1	-25.200	-140.853	90.452	-17.065	-116.247	82.117	0.909	0.911

Supplementary Table 3. Changes of DNA methylation-based aging markers and surrogate markers of GrimAge during intervention (mean ± SD).

		INT	CON
PC-PhenoAge	Week 0	-1.13 ± 2.87	0.92 ± 3.66
	Week 12	-1.28 ± 3.54	0.63 ± 4.10
PC-HorvathClock	Week 0	-0.62 ± 2.60	1.07 ± 2.22
	Week 12	-0.30 ± 2.53	1.43 ± 2.36
PC-HannumClock	Week 0	-0.70 ± 2.32	0.59 ± 2.09
	Week 12	-0.51 ± 2.65	0.98 ± 2.21
GrimAge	Week 0	0.90 ± 3.36	2.95 ± 3.47
	Week 12	0.70 ± 3.19	3.41 ± 3.65
GrimAgev2	Week 0	0.43 ± 3.89	2.81 ± 3.54
	Week 12	0.47 ± 3.77	3.18 ± 3.70
DunedinPACE	Week 0	1.05 ± 0.08	1.10 ± 0.07
	Week 12	1.03 ± 0.09	1.10 ± 0.07
DNAmADM	Week 0	1.45 ± 21.58	-2.38 ± 16.76
	Week 12	1.33 ± 19.34	-1.22 ± 18.72
DNAmB2M	Week 0	-12803.01 ± 43146.48	1415.11 ± 50947.56
	Week 12	-16580.52 ± 29680.02	6862.68 ± 39378.58
DNAmCystatinC	Week 0	-403.69 ± 15965.27	3462.10 ± 16561.07
	Week 12	-3922.31 ± 11831.65	8084.86 ± 12774.48
DNAmGDF15	Week 0	-3.04 ± 64.60	37.17 ± 46.00
	Week 12	-5.50 ± 53.38	37.38 ± 52.06
DNAmLeptin	Week 0	-219.47 ± 1417.09	326.89 ± 1611.20
	Week 12	-583.42 ± 1068.03	-281.05 ± 1181.55
DNAmPAI1	Week 0	-468.07 ± 2117.31	-496.06 ± 2263.30
	Week 12	-690.36 ± 2072.97	-467.18 ± 2216.02
DNAmTIMP1	Week 0	-71.97 ± 448.88	4.77 ± 388.15
	Week 12	-71.55 ± 455.30	3.58 ± 352.49
