# Supplementary material for: Short-term responsiveness of DNA methylation–based aging biomarkers to a multimodal intervention comprising exercise and dietary guidance involving daily consumption of yogurt containing Bifidobacterium longum BB536: an exploratory randomized controlled trial
Source: Aging (Albany NY). 2026 May 29;18(1):639–55. doi: 10.18632/aging.206386 (PMC13632858; doi:10.18632/aging.206386)
Supplement: Supplementary file 3 [file aging-18-1-206386-s004.pdf]

Supplementary Table 5. Frequency of exercise sessions per week during the intervention.

Donor of INT group	Mean ± SD (sessions/week)
ID01	5.3 ± 1.3
ID02	6.9 ± 0.3
ID03	6.2 ± 1.0
ID04	5.8 ± 1.4
ID05	4.3 ± 1.8
ID06	6.8 ± 0.5
ID07	3.1 ± 0.3
ID08	5.1 ± 1.1
ID09	3.8 ± 2.0
ID10	7.0 ± 0.0
ID11	5.7 ± 1.1
ID12	4.8 ± 1.3
ID13	6.8 ± 0.5
ID14	3.0 ± 0.9
ID15	4.8 ± 1.0
ID16	5.3 ± 1.5
ID17	3.5 ± 0.7
ID18	4.8 ± 1.2
ID19	3.9 ± 1.1
ID20	6.8 ± 0.5
ID21	5.6 ± 1.1
Average	5.2 ± 1.3
