# Supplementary material for: Short-term responsiveness of DNA methylation–based aging biomarkers to a multimodal intervention comprising exercise and dietary guidance involving daily consumption of yogurt containing Bifidobacterium longum BB536: an exploratory randomized controlled trial
Source: Aging (Albany NY). 2026 May 29;18(1):639–55. doi: 10.18632/aging.206386 (PMC13632858; doi:10.18632/aging.206386)
Supplement: Supplementary file 4 [file aging-18-1-206386-s003.docx]

Supplementary Table 4. Participant characteristics at baseline and changes in body composition and mood state over 12 weeks.

| Group | ID | Chronological age (years) | Current smoker (Yes/No) | Sleep duration (h) | Body weight (kg) | | BMI (kg/m^2^) | | POMS 2 TMD score | |
| --- | --- | --- | --- | --- | --- | --- | --- | --- | --- | --- |
|  |  |  |  |  | Week 0 | Week 12 | Week 0 | Week 12 | Week 0 | Week 12 |
| INT | ID01 | 53 | No | 5 | 76.5 | 77.8 | 25.6 | 26.1 | 42 | 43 |
| INT | ID02 | 65 | No | 8 | 83.4 | 79.7 | 27.1 | 25.9 | 54 | 52 |
| INT | ID03 | 55 | No | 8 | 100.8 | 95.5 | 33.1 | 31.4 | 50 | 54 |
| INT | ID04 | 52 | No | 6 | 78.6 | 76.8 | 25.3 | 24.7 | 53 | 58 |
| INT | ID05 | 58 | No | 8 | 79.1 | 78.8 | 25.9 | 25.8 | 48 | 51 |
| INT | ID06 | 56 | No | 6 | 82.8 | 77.4 | 26.3 | 24.6 | 40 | 39 |
| INT | ID07 | 54 | No | 6 | 79.5 | 78.7 | 27.8 | 27.5 | 49 | 47 |
| INT | ID08 | 56 | No | 6 | 83.7 | 80.4 | 27.1 | 26.0 | 42 | 47 |
| INT | ID09 | 61 | Yes | 4.5 | 90.1 | 85.9 | 35.0 | 33.3 | 38 | 37 |
| INT | ID10 | 51 | Yes | 6.5 | 74.5 | 71.8 | 26.1 | 25.1 | 39 | 42 |
| INT | ID11 | 68 | No | 7 | 76.1 | 74.3 | 25.6 | 25 | 39 | 40 |
| INT | ID12 | 55 | No | 7 | 86.8 | 85.7 | 26.1 | 25.7 | 36 | 37 |
| INT | ID13 | 54 | No | 7 | 69.7 | 68.5 | 26.1 | 25.7 | 45 | 40 |
| INT | ID14 | 62 | No | 5 | 91.2 | 90.0 | 27.5 | 27.1 | 51 | 57 |
| INT | ID15 | 58 | No | 8 | 90.1 | 89.2 | 28.8 | 28.5 | 48 | 46 |
| INT | ID16 | 52 | Yes | 7 | 71.7 | 67.4 | 25.8 | 24.2 | 41 | 38 |
| INT | ID17 | 59 | No | 6 | 79.1 | 72.1 | 27.7 | 25.3 | 40 | 41 |
| INT | ID18 | 58 | No | 6 | 90.5 | 85.2 | 31.4 | 29.6 | 49 | 46 |
| INT | ID19 | 61 | No | 6 | 91.3 | 88.2 | 29.5 | 28.5 | 40 | 36 |
| INT | ID20 | 59 | Yes | 6 | 91.6 | 86.9 | 32.2 | 30.5 | 44 | 40 |
| INT | ID21 | 58 | No | 7 | 84.8 | 80.3 | 25.9 | 24.6 | 39 | 36 |
| CON | ID22 | 54 | Yes | 7 | 101.9 | 101.1 | 32.4 | 32.2 | 42 | 44 |
| CON | ID23 | 57 | No | 5 | 97.8 | 93.6 | 32.3 | 30.9 | 40 | 43 |
| CON | ID24 | 68 | Yes | 7 | 72.2 | 72.4 | 25.5 | 25.6 | 33 | 33 |
| CON | ID25 | 62 | No | 6 | 75.2 | 75.0 | 26.5 | 26.4 | 37 | 38 |
| CON | ID26 | 54 | No | 4.5 | 69.2 | 67.8 | 25.3 | 24.8 | 64 | 63 |
| CON | ID27 | 58 | No | 6 | 87.5 | 87.7 | 29.5 | 29.6 | 43 | 41 |
| CON | ID28 | 56 | Yes | 8 | 86.5 | 85.9 | 32.7 | 32.5 | 41 | 41 |
| CON | ID29 | 65 | No | 7 | 72.2 | 72.1 | 25.7 | 25.7 | 36 | 33 |
| CON | ID30 | 58 | No | 7.5 | 83.1 | 84.5 | 26.6 | 27.1 | 48 | 49 |
| CON | ID31 | 59 | Yes | 8 | 88.3 | 86.9 | 26.0 | 25.6 | 38 | 45 |
| CON | ID32 | 59 | No | 7 | 77.4 | 76.5 | 27.6 | 27.3 | 46 | 48 |
| CON | ID33 | 50 | Yes | 6 | 75.8 | 78.5 | 26.6 | 27.6 | 49 | 48 |
| CON | ID34 | 57 | No | 6 | 83.0 | 81.4 | 28.0 | 27.4 | 41 | 42 |
| CON | ID35 | 62 | No | 5 | 75.6 | 68.3 | 26.3 | 23.7 | 41 | 43 |
| CON | ID36 | 54 | No | 6 | 87.7 | 89.1 | 26.7 | 27.2 | 35 | 52 |
| CON | ID37 | 51 | Yes | 7 | 74.7 | 75.6 | 25.7 | 26.0 | 55 | 48 |
| CON | ID38 | 53 | Yes | 7 | 76.6 | 76.4 | 25.4 | 25.3 | 42 | 45 |
| CON | ID39 | 62 | No | 8 | 83.8 | 85.4 | 25.7 | 26.2 | 43 | 47 |
| CON | ID40 | 54 | No | 5 | 69.0 | 70.8 | 25.2 | 25.8 | 42 | 41 |
| CON | ID41 | 61 | No | 6 | 82.7 | 83.9 | 25.9 | 26.3 | 51 | 45 |
| CON | ID42 | 56 | Yes | 5 | 88.6 | 88.5 | 32.5 | 32.4 | 52 | 55 |
| CON | ID43 | 59 | No | 7 | 81.5 | 79.7 | 28.5 | 27.8 | 65 | 62 |
| CON | ID44 | 51 | No | 7 | 81.0 | 80.8 | 28.8 | 28.7 | 55 | 60 |
| CON | ID45 | 71 | No | 7 | 75.4 | 75.8 | 28.4 | 28.6 | 44 | 46 |
